# Oral Health-related Quality of Life and Daytime Sleepiness in Students

**DOI:** 10.3290/j.ohpd.c_1873

**Published:** 2025-04-10

**Authors:** Cristian Sánchez Vaca, Juan Ignacio Rosales Leal, Rocío Barrios-Rodríguez

**Affiliations:** a Cristian Sánchez Vaca Dentist and PhD Student, Department of Prosthodontics and Orofacial Pain, School of Dentistry, University of Granada, Spain. Idea, study design, data collection, wrote the manuscript.; b Juan Ignacio Rosales Leal Dentist and Professor, Department of Prosthodontics and Orofacial Pain, School of Dentistry, University of Granada, Spain. Idea, study design, proofread the manuscript, proofread statistical evaluation.; c Rocío Barrios-Rodríguez Dentist and Professor, Instituto de Investigación Biosanitaria ibs.GRANADA, Complejo Hospitales Universitarios de Granada/Universidad de Granada, Spain; Consortium for Biomedical Research in Epidemiology and Public Health (CIBERESP), Madrid, Spain. Department of Preventive Medicine and Public Health, University of Granada, Granada, Spain. Statistical evaluation.

**Keywords:** daytime sleepiness, dental students, oral health impact profile, oral health-related quality of life.

## Abstract

**Purpose:**

This study aimed to investigate the association between excessive daytime sleepiness and oral health-related quality of life (OHRQoL) in dental and dental-hygiene students using a cross-sectional design.

**Materials and Methods:**

Dental students and dental-hygiene students completed a sociodemographic and habits questionnaire. Oral health-related quality (OHRQoL) of life was assessed using the Oral Health Impact Profile (OHIP), and sleep quality was determined using the Epworth Sleepiness Scale. Student’s t-tests and chi-squared tests were used to analyse the association between oral health-related quality of life with sleep and other variables. A linear regression model was constructed to adjust the effect of daytime sleepiness for oral health-related quality of life.

**Results:**

The dimenstion of the OHIP that had the worst scores was physical pain (mean: 1.45; SD: 1.49). Excessive daytime sleepiness impaired the OHRQoL specifically because of physical pain, functional limitation, physical disability, and psychological disability. In the adjusted analysis, the presence of excessive daytime sleepiness increased the OHIP score to 2.54 points (95% CI: 1.09-3.99). To be female and to study at the technical-school level was also associated with a reduction of OHRQoL.

**Conclusion:**

The presence of excessive daytime sleepiness impaired the OHRQoL of students. Excessive daytime sleepiness is a factor associated with lower OHRQoL. Integration of sleep monitoring in interventions aimed at improving the OHRQoL could have a relevant impact on young adult patients.

In Spain, sleep disorders have a prevalence of 38% in the general population, being more prevalent in women (44.6%) than in men (30.1%).^20^ The International Classification of Sleep Disorders includes insomnia, sleep-related breathing disorders, central disorders of hypersomnolence, circadian rhythm sleep-wake disorders, parasomnia, sleep-related movement disorders, and other sleep disorders,^28^ which have in common that they negatively affect resting. Among the central disorders of hypersomnolence, daytime sleepiness is defined as the propensity of an individual to fall asleep in situations where other people would not^15^ and is an expression of a sleep disorder. The prevalence of daytime sleepiness changes according to the population studied, and ranges between 18.0 and 50.8%.^7,12,13,25,32^

Sleep deficits are significantly and substantially associated with a variety of negative consequences. The effects of sleep deprivation include reduced ability to pay attention, lower psychomotor surveillance, and greater variability in behavioural responses, effects that are associated with impaired functioning of the prefrontal dorsolateral cortex and parietal regions.^17^ Different studies have suggested that excessive daytime sleepiness is related to Parkinson’s disease, tension headache, or Crohn’s disease.^36,18,3^ Daytime sleepiness can be predicted by stress, which may affect sleep quality through several manifestations, such as anxiety.^37^

Moreover, sleep disorders have been associated with oral health issues.^4,27^ Some findings provide evidence for an association between sleep disorders and gingival inflammation.^4^

It is well known that periodontal health is fundamental to an individual’s overall health. In vulnerable individuals, periodontal disease extends beyond oral health, impacting various organs. It is associated with systemic diseases, particularly in conditions like diabetes, metabolic syndrome, obesity, liver and cardiovascular diseases, Alzheimer’s, rheumatoid arthritis, pregnancy complications, and certain cancers.^16^ These associations are not only bidirectional but also multimodal, involving multiple comorbidities.^16^ Other research^2^ indicates a significant association between stress, mental health disorders, and periodontal disease. Conditions such as anxiety, depression, bipolar disorder, schizophrenia, Alzheimer’s disease, and substance use disorders are linked to more severe periodontal disease and, in some instances, reduced healing outcomes following nonsurgical periodontal therapy.^2^

However, the objective parameters of oral health do not always correspond to how subjects feel or how they adapt to their circumstances.^34^ An interest in analysing subjective parameters of oral health such as oral health-related quality of life (OHRQoL) has increased in recent years. OHRQoL assessments provide valuable information on oral health from the patient’s subjective point of view. OHRQoL evaluation is particularly important in young people, as this might be more subjectively impaired at that age due to high expectations and less satisfaction with health aspects,^31^ and because young people represent a critical age group with specific oral health needs.^1^

Recent studies suggest an association between sleep disorders and OHRQoL in other populations.^10,24^ However, to our knowledge, there are no studies in young people focused on daytime sleepiness and OHRQoL that could associate both parameters. This study is motivated by the growing body of evidence linking sleep disorders with both oral and systemic health, particularly in younger populations. Given the critical role of sleep in overall health, it is crucial to explore the potential impact of excessive daytime sleepiness on OHRQoL. Therefore, the objective of this study is to assess the relationship between these two variables and address the existing gap in the literature concerning young adults. The aim of this study was to evaluate the relationship between OHRQoL and daytime sleepiness in dental students and dental hygiene students.

## MATERIALS AND METHODS

A survey was carried out on first, second and third year students (n = 118, n = 85 and n = 70 respectively) of the School of Dentistry at the University of Granada (Spain) as well as students in their final year of training at a private technical school to become a dental hygienist (n = 69). After explanation of the objective of study, surveys were administered to those students that volunteered to participate. The questionnaires were self-administered, anonymous, and could be completed without the interviewer’s intervention. Although this study did not include clinical measurements, the use of validated questionnaires allowed the assessment of subjective experiences of OHRQoL and daytime sleepiness, which are crucial for understanding patients’ perceptions.

The first part of the survey collected sociodemographic data and variables related to lifestyle: age, sex, tobacco use (never, ex-smoker, current smoker), alcohol, drugs, coffee and physical exercise (where response options were: never, rarely, occasionally 1-2 times a week, every day), presence of chronic diseases (diabetes, thyroid disease, epilepsy, allergies or blood pressure problems) at the time of the interview, and whether they had children.

Daytime sleepiness was measured with the Epworth Sleepiness Scale (Spanish validated version^8^). The patients answered items about 8 common daily situations with a score ranging from 0 to 3, from least to greatest: 0 = would never doze; 1 = slight chance of dozing; 2 = moderate chance of dozing; 3 = high chance of dozing. These scores were summed and grouped. Thus, patients were classified into: 0–5 = low normal daytime sleepiness; 6–10 = increased normal daytime sleepiness; 11–12 = mild excessive daytime sleepiness; 13–15 = moderate excessive daytime sleepiness; 16–24 = severe excessive daytime sleepiness.

The OHRQoL was assessed using the Spanish validation version of OHIP-14 questionnaire.^23^ OHIP-14 has seven dimensions (each one with two questions): functional limitation, physical pain, psychological discomfort, physical disability, psychological disability, social disability and disability. Patients responded according to the frequency of oral impacts on a 5-point Likert scale as follows: never (0), almost never (1), occasionally (2), quite frequently (3) and very often (4), taking into account the last 12 months. In order to calculate the total and dimensions scores, the additive method OHIP-AD was used, adding up the scores obtained in the two items of each dimension and the 14 items respectively. Higher scores indicated worse OHRQoL.

The statistical analysis was done with SPSS v.24 (Statistical Package for Social Sciences). For bivariate analyses, the distribution of different variables regarding OHRQoL was assessed using Student’s t-test, Pearson’s correlation coefficient and ANOVA. The difference in OHRQoL between students with excessive daytime sleepiness and students with no excessive daytime sleepiness was analysed. Furthermore, to assess the clinical importance of these differences, we calculated the effect sizes.^35^ The interpretation was done according to the benchmarks suggested by Cohen’s standard criteria.^5^ The linear regression model was constructed to evaluate the association between excessive daytime sleepiness and OHRQoL. Taking into account epidemiological and statistical criteria, the model was adjusted for sex, level of education, coffee intake and whether or not the particpant had children. Statistical significance was set at p < 0.05.

## Results

Sociodemographic and lifestyle data and their relation to OHRQoL are shown in Table 1. 74.9% of students were women, and the mean age of students was 20.8 (SD: 4.9) years. The majority of the sample were university students (79.3%). Only 6.3% had children and 41.3% had a somatic or chronic disease at the time of the study. Regarding the lifestyle variables, 78.1% did not smoke, 80.4% drank alcohol occasionally or at least once a week, and 92.8% did not take other drugs. Physical exercise was done occasionally by 70.1% of the students, and only 19.2% practiced it every day. The variables statistically significantly associated with worse OHRQoL were being female (a mean of 7.4 [SD: 7.1] vs a mean of 5.4 [SD: 5.4] in males) and to study at a technical school (mean 8.6 [SD: 6.6] vs mean 6.5 [SD: 6.7] in university students).

**Table 1:** Association between sociodemographic characteristics/lifestyles and oral health-related quality of life (OHRQoL) (n = 334)

Variable	n (%)	OHRQoL^a^ mean (SD)	p-value
Gender Male Female	84 (25.1) 250 (74.9)	5.4 (5.4) 7.4 (7.1)	0.015^b^
Age (years), mean ± SD	20.8 ± 4.9	6.9 (6.8)	0.237^c^
Education level Technical school University	69 (20.7) 265 (79.3)	8.6 (6.6) 6.5 (6.7)	0.025^b^
Have children No Yes	313 (93.7) 21 (6.3)	6.7 (6.6) 9.6 (8.3)	0.059^b^
Presence of diseases No Yes	196 (58.7) 138 (41.3)	6.9 (6.9) 7.0 (6.5)	0.814^b^
Tobacco consumption No Ex-smokers Current smokers	261 (78.1) 30 (9.0) 43 (12.9)	6.8 (0.4) 7.7 (1.0) 7.3 (1.2)	0.728^d^
Alcohol intake No Occasionally At least once a week	62 (18.6) 168 (50.3) 104 (30.1)	7.0 (1.0) 6.6 (0.4) 7.4 (0.8)	0.689^d^
Other drugs No Occasionally	310 (92.8) 24 (7.2)	6.8 (6.8) 6.2 (1.3)	0.236^b^
Coffee No Occasionally Every day	76 (22.8) 132 (39.5) 126 (37.7)	6.2 (0.6) 6.4 (0.5) 8.0 (0.7)	0.105^d^
Physical exercise No Occasionally Every day Missing	19 (5.7) 240 (75.1) 64 (19.2) 11	7.5 (1.7) 7.1 (0.4) 5.7 (0.9)	0.323^d^
^a^Measurement with Oral Health Impact Profile-14 questionnaire; ^b^Student’s t-test; ^c^Pearson’s correlation coefficient; ^d^ANOVA.

Table 2 shows the description of dimensions of OHRQoL and its association with daytime sleepiness. The most impairing dimension in the sample was physical pain (mean: 1.45; SD: 1.49), followed by functional limitation (mean: 1.42; SD 1.24). Excessive daytime sleepiness was associated with worse functional limitation, physical pain, physical and psychological disability. Students with excessive daytime sleepiness had a worse total OHIP-14 score than students without (means 5.91 and 8.42 for the no excessive sleepiness group and daytime sleepiness group, respectively). This difference was clinically relevant with a moderate effect size (0.38; 95% CI: 0.14-0.63).

**Table 2:** Description of dimensions of OHIP-14^a^ and its association between oral health-related quality of life and sleep quality of students

OHIP-14 dimensions	Total (n = 334) mean ± SD	No excessive daytime sleepiness (n = 199) mean ± SD	Excessive daytime sleepiness (n = 135) mean ± SD	p- value^b^
Functional limitation	1.42 ± 1.24	1.26 ± 1.11	1.67 ± 1.39	0.013
Physical pain	1.45 ± 1.49	1.30 ± 1.41	1.66 ± 1.59	0.029
Psychological discomfort	1.19 ± 1.60	1.03 ± 1.39	1.42 ± 1.85	0.118
Physical disability	1.02 ± 1.53	0.83 ± 1.36	1.28 ± 1.72	0.042
Psychological disability	0.79 ± 1.47	0.64 ± 1.33	1.01 ± 1.63	0.011
Social disability	0.73 ± 1.46	0.60 ± 1.29	0.93 ± 1.67	0.073
Handicap	0.35 ± 0.95	0.28 ± 0.77	0.46 ± 1.16	0.090
OHIP summary score^c^	6.93 ± 6.76	5.91 ± 5.95	8.42 ± 7.56	<0.001
^a^OHIP-14: Oral Health Impact Profile-14 questionnaire; ^b^Student’s t-test; ^c^The effect size of the difference was 0.38 (95% CI: 0.14-0.63).

A linear regression analysis was carried out just to adjust for the association between daytime sleepiness and OHRQoL and its results are shown in Table 3. The OHRQoL remained associated with daytime sleepiness: students with excessive daytime sleepiness had an OHIP-14 score of 2.54 (95% CI: 1.09-3.99). Similar to bivariate analysis, gender and type of tertiary education (technical school vs university) were variables associated with OHRQoL, although gender was not statistically significant.

**Table 3:** Linear regression analysis with oral health-related quality of life as dependent variable (n = 334)

Variable	OHIP-14^a^ β (95% CI)	p-value
Sleep quality No excessive daytime sleepiness Excessive daytime sleepiness	1.00 2.54 (1.09, 3.99)	0.001
Sex Men Women	1.00 1.59 (-0.05, 3.22)	0.057
Education level University Technical school	1.00 1.98 (0.19, 3.77)	0.030
Coffee No/occasionally Every day	1.00 0.65 (-0.29, 1.59)	0.176
Have children No Yes	1.00 2.03 (-0.96, 5.01)	0.182
^a^OHIP-14: Oral Health Impact Profile-14 questionnaire.

## DISCUSSION

The objective of this study was to analyse the OHRQoL of dental students and dental-hygiene students and its association with sleep characteristics. The most relevant result of this study was that daytime sleepiness negatively affected OHRQoL. It was found that physical pain was the dimension that most impaired OHRQoL and students with excessive daytime sleepiness had worse scores in that dimension. Moreover, there were clinically relevant differences in overall OHRQoL between students with excessive daytime sleepiness and students without. This association remained in the adjusted model, where excessive daytime sleepiness increased the OHIP-14 to 2.54 points. Gender and type of tertiary education (technical school vs university) were also variables related to the OHRQoL of our sample.

This study presents potential limitations. Although it is appropriate to the proposed objectives, the cross-sectional design of the study does not allow cause/effect relationships to be established. Only dental hygiene students and dental students participated in the study, which may limit the generalisability of our findings. However, there was a high response rate (100% dental hygienist students and 97.1% of dental students), which creates a well-characterised group and allows postulating new hypotheses from these results. In this study, only a questionnaire without clinical examination was used, which could be a limitation. However, in this way it was possible to reach a large and anonymous sample. In addition, the use of standardised and validated questionnaires allows us to compare the results with other studies.

Physical pain was the OHRQoL dimension with the worst score among students (mean: 1.45; SD: 1.49). This finding is consistent with the literature. Previous studies have found physical pain as the dimension most frequently reported^6^ or most impairing in students.^9,25^ Moreover, this study found that excessive daytime sleepiness was associated with worse scores in that dimension. This could be because the lack of sleep reduces tolerance to pain, and much more so if the pain is chronic.^30^ The relationship between pain and lack of sleep is based on two neurobiological aspects: 1) A lack of sleep reduces serotonin levels, reducing the activity of the descending inhibitory system, which translates into a greater perception of pain^19^ and 2) the system of endorphins, which act as natural analgesics, are reduced in people with poor sleep quality.^19^

The overall OHIP-14 score was also greatly influenced by daytime sleepiness, with a moderate effect size of the score difference between students with excessive daytime sleepiness and students without (mean difference: 2.51; ES: 0.38, 95% CI 0.14-0.63). This association remained in the adjusted model, increasing the OHIP-14 scores to 2.54 points when excessive daytime sleepiness was present. Thus, the presence of this sleep disorder could have relevant clinical implications for OHRQoL. In this sense, oral clinical examination may include a routine evaluation of sleep disorders to better manage patients’ OHRQoL and dental treatments.^29^

The OHRQoL was also affected by the level of education. Educational level had a positive association in the adjusted analysis, which may be due to increased self-awareness with increasing education.^21^ Other studies concluded that a clear education gradient existed in OHRQoL, with worse perceptions correlated with a lower level of education.^22,33^ The students from the technical school, with a lower OHRQoL despite studying dental hygiene, represent a population less careful with their oral health, which implies a worse OHRQoL.

Emerging evidence suggests that work-related stress may exacerbate periodontal diseases, particularly in high-stress occupations. Stress-related occupations have been linked to a higher prevalence of periodontitis, which may further impact the overall quality of life. Addressing occupational stress in dental and general health professionals could mitigate the progression of periodontal disease and improve patient outcomes.

Although it was not statistically significant, sex tended to be associated with OHIP-14 scores, with more impaired OHRQoL among women. The results in other studies are contradictory. In some cases, there are no differences between men and women,^21^ and others conclude that young women have a better OHRQoL.^11^ The fact that women have a worse OHRQoL could be due to the fact that men have less awareness about their appearance and they answer the survey as if they had a better OHRQoL than they really do.^14^

With the limitations of this study, our findings support the hypothesis that excessive daytime sleepiness statistically significantly impairs OHRQoL. Notably, the presence of excessive daytime sleepiness was associated with a statistically significant decline in both the physical and psychological dimensions of OHRQoL, further corroborating previous research on the negative health impacts of poor sleep. The gender and level of education was also associated with OHRQoL. Being female and having a lower education level are related to lower OHRQoL. Although more studies are needed to confirm these results, integration of sleep evaluations in oral health examinations could have a relevant impact on improving the OHRQoL. Besides, these findings hold important clinical relevance, suggesting that monitoring and addressing sleep disorders such as excessive daytime sleepiness could play a critical role in improving not only oral health outcomes but also the overall quality of life in young adults. Future research should explore interventions that target sleep disorders as part of comprehensive oral health management strategies.
